# Investigation and Analysis of Anxiety and Quality of Life among Systemic Lupus Erythematosus Patients in Northwestern China

**DOI:** 10.3390/healthcare10112180

**Published:** 2022-10-31

**Authors:** Qing Wang, Junfeng Jia, Kui Zhang, Zhaohui Zheng, Huilin Liu

**Affiliations:** 1 Department of Clinical Immunology, Xijing Hospital, Fourth Military Medical University, Xi’an 710000, China; 2 Department of Rehabilitation Medicine, Xijing Hospital, Fourth Military Medical University, Xi’an 710000, China

**Keywords:** systemic lupus erythematosus, questionnaire survey, quality of life, anxiety

## Abstract

The aim of this study was to provide targeted psychological support and effective nursing for systemic lupus erythematosus (SLE) patients. SLE is a complex, systemic autoimmune disease characterized by recurrent episodes and the involvement of multiple organs. With improvements in SLE treatment and the corresponding increase in patients’ survival time, the quality of life (QoL) of SLE patients has become an important indicator for evaluating the effectiveness of clinical treatments. To explore the anxiety states and health-related QoL of SLE patients, 106 SLE patients were asked to provide responses for the short-form 36 health survey (SF36), and the Systemic Lupus Erythematosus Disease Activity Index (SLEDAI) and Visual Analog Scale(VAS). Additionally, the Systemic Lupus Collaborative Clinics Damage Index (SDI) was analyzed. Data regarding patients’ age, gender, education level, occupation, family income, and duration of disease were collected. Regression analysis was performed to identify factors related to patients’ health-related QoL. For the SF36, the mental components score (MCS), mental health (MH), and bodily pain (BP) occupied dominant positions. Additionally, the MH domain was significantly associated with anxiety in SLE patients. Negative relationships were identified between irregular sleep and the scores for role limitations due to physical problem (RP), vitality (VT), and role limitations due to emotional problem (RE) domains. From the analysis of SLEDAI and SDI scores, anxiety among SLE patients was mainly affected by disease activity and quality of life. This study provides a preliminary understanding of the QoL of SLE patients in western China and highlights the need for the future development of strategies to provide targeted psychological support and effective nursing for SLE patients, in order to improve patients’ self-awareness, mental health, and QoL.

## 1. Introduction

Systemic lupus erythematosus (SLE) is a chronic autoimmune disease characterized by recurrent episodes and the involvement of multiple organs, including the skin, joints, kidneys, nervous system, and serous membranes [1,2]. These effects can influence patients’ abilities to perform activities of daily living, abilities to work, and overall quality of life (QoL) [3]. It is well established that the disease activity varies in different individuals over time, and subjective symptoms can become prominent factors [4,5]. Therefore, researchers have focused on some predefined aspects of SLE, such as the disease impact and selected symptoms, in developing the existing standards for assessing the degree of disease. Although clinical nurses have gradually noticed the impact of psychological factors on patients with this disease in the course of treating patients with SLE, there remains a lack of early and comprehensive methods for support and intervention. Thus, there is a need for the development of targeted nursing measures and to highlight the role of nursing work in the management of patients with chronic diseases. Additional clinical research is necessary to identify the anxiety status of SLE patients as early as possible and implement early psychological care [6,7].

Health-related quality of life (HRQoL) is an important indicator for measuring disease activity and damage [8,9,10]. In recent years, more attention has been given to the relationship between HRQoL and SLE management in addition to therapies for the disease and organ damage [11], and HRQoL assessments have become a part of patient care since the publication of the Outcome Measures in Rheumatology Clinical Trials (OMERACT) group recommendations in 2000 [12]. Previous studies have shown that the QoL of patients with SLE is often very poor [13,14]. Extensive body damages and relevant visible characteristics can be found among patients with SLE and may be caused by multiple factors [15]. More than two-thirds of SLE patients experience disease characteristics that directly affect QoL, such as facial erythema, pathologic pigmentation, light sensitivity, hair loss, skin ulcers, and muscle fiber pain. The manifestation of these physical symptoms will not only cause physical harm to patients with SLE but also cause serious trauma to their psychology, which will affect their attitude toward life and QoL. Meanwhile, behavioral factors and mood, as well as sleep disorders, may play important roles in the attitudes toward life of patients with SLE [16]. Despite great improvements in the prognosis of patients with SLE in recent decades, HRQoL remains compromised in such patients when compared with healthy controls and patients affected by other chronic diseases. Accordingly, anxiety is a common manifestation of SLE patients. It has been reported that the prevalence of anxiety disorders in SLE patients is two times higher than that in the general population [17]. In China, the reported prevalence of anxiety symptoms in SLE patients is as high as 86.8% [18]. Anxiety can impact SLE patients’ health and induce various complications [19]. Therefore, treatment of anxiety can be an important goal to improve the QoL of SLE patients. Toward this goal, studies are beginning to investigate the etiology, influence, and management of QoL and anxiety in SLE patients. However, compared with research related to other similar chronic diseases, there is still a paucity of data for SLE. Therefore, more related research is needed to understand the interaction between SLE patients’ mental and physical health and the effects of SLE therapies to provide better guidance for further management and intervention [20].

Therefore, in order to investigate the relationships among psychological status, QoL, and anxiety in SLE patients, we conducted a questionnaire survey in 106 SLE patients in western China and analyzed the relationships between anxiety, disease activity, QoL, and other factors. The results obtained provide a basis for applying corresponding psychological intervention measures in clinical practice.

## 2. Patients and Methods

### 2.1. Patients

A total of 106 patients who were admitted to the Department of Clinical Immunology of Xijing Hospital from October 2017 to October 2018 were selected for investigation if diagnosed according to either the 1997 American College of Rheumatology Classification Criteria for Systemic Lupus Erythematosus [21] or the classification criteria for systemic lupus established by Systemic Lupus International Collaborating Clinics in 2012 [22]. Patients aged less than 18 years and those with primary diabetes, kidney disease, malignant tumors, or mental illness were excluded. Patients with invalid questionnaires were also excluded.

### 2.2. Study Design

The questionnaire included the basic information of the patient, for example, gender, age, ethnicity, occupation, marital status, fertility, education, course of illness, number of hospitalizations, symptoms, disease awareness, sleep quality, etc. The patients also provided responses for the short-form 36 health survey (SF36), the Systemic Lupus Erythematosus Disease Activity Index (SLEDAI), the Systemic Lupus Collaborative Clinics Damage Index (SDI), and Visual Analog Scale(VAS) during a clinical visit.

The QoL self-assessment scale is an international scale for measuring subjective HRQoL that was prepared and recommended by the World Health Organization Quality of Life Questionnaire Development Group in 1996. It includes four fields (the physiological field, psychological field, social relationship field, and environmental field) and two independent analysis items (the individual’s aggregate subjective feelings about their own QoL and the individual’s total subjective feelings about their own health). The field scores are scored in the forward direction (the higher the score, the better the QoL). The field score is obtained by calculating the average score of the item to which it belongs and multiplying by 4. The score results for each field are summed to obtain the WHOQO L-100 scale [23].

To assess anxiety, we used the self-assessment scale of anxiety (SAS) compiled by Zung [24]. The scale consists of 20 items and is appropriate for adults with anxiety symptoms and for investigating the frequency of anxiety symptoms. In Chinese patients, the cut-off value for anxiety on the SAS is 50 points, with a score of 50–59 indicating mild anxiety, a score of 60–69 indicating moderate anxiety, and a score of >70 points reflecting severe anxiety.

Disease activity was evaluated using the SLEDAI scoring software for all patients admitted to the hospital and those receiving treatment [25]. On the SLEDAI scale, a score of <4 indicates remission, 5–9 corresponds to low disease activity, 10–14 indicates moderate disease activity, and ≥15 reflects high disease activity.

Patients’ pain was examined using the VAS, an established scale for assessing pain.

### 2.3. Statistical Analysis

Scores were obtained for all included patients by designated personnel using unified guidelines. The trained investigators explained all information related to the surveys to each patient, and then the patients completed the self-evaluations. If the questionnaire could be conducted independently, the investigator conducted a face-to-face interview in which the questionnaire was completed. SPSS version 11.0 statistical software (SPSS Inc., Chicago, IL, USA) was utilized to analyze the correlations among the investigated dimensions and factors [26]. *p* < 0.05 presented significant differences between samples. In addition, multivariate logistic regression was used to analyze the relationships between the quality of life in SLE patients and sleep quality, SLEDAI and SDI scores, and anxiety levels, using the patient’s sleep as the dependent variable and the content in the SF-36 statistical table as the independent variable.

## 3. Results

### 3.1. Patient Characteristics

We received a total of 106 completed questionnaires, with a survey completion rate of 100%. The basic characteristics of the included SLE patients are presented in Table 1. The proportion of female patients (97%) was much greater than that of male patients (9%). The age range of 41–50 years included the most patients, accounting for 29.25% of the total number. The ratio of urban to rural patients was close to 1:1. The survey of education level also showed that all levels were represented among the patients, but most had graduated from high school or technical secondary school. In addition, important factors such as medical expense payment method and income were included in the survey. Most of the patients belonged to middle-income families, and they mainly received rural cooperative medical service. The mean patient age was 44.2 ± 11.40 years, and the mean disease duration was 18.5 ± 9.21 years. The mean SLEDAI score was 4.00 ± 4.70, and the mean SDI score was 1.5 ± 4.3.

### 3.2. Measures of Eight Domains in SF-36

For the study population, the mean scores for the domains of the SF-36 were 73.4 ± 21.5 for physical functioning (PF), 69.4 ± 27.7 for role limitations due to physical problems (RP), 62.9 ± 25.7 for bodily pain (BP), 49.2 ± 20.1 for general health (GH), 53.1 ± 21.7 for vitality (VT), 71.2 ± 24.7 for social functioning (SF), 75.1 ± 25.6 for role limitations due to emotional problems (RE), and 62.6 ± 14.8 for mental health (MH). However, norm-based scores are more commonly used for the SF-36 rather than the original scores, according to the 0–100 score. These scores for each domain were 37.9 ± 18.1 for PF, 39.7 ± 15.4 for RP, 46.2 ± 12.7 for BP, 42.1 ± 11.4 for GH, 45.3 ± 11.2 for VT, 42.7 ± 13.4 for SF, 42.9 ± 13.5 for RE, and 46.8 ± 9.3 for MH. The mean scores for the two summary measures are 39.2 ± 15.6 for the physical component score (PCS) and 47.9 ± 9.8 for the mental component score (MCS).

### 3.3. Factors Associated with QoL in SLE Patients

Analysis of the relationships between clinical factors and the components of the SF-36 showed that poor sleep was significantly negatively associated with RP (adjusted odds ratio (OR) = 8.32, *p* = 0.011), VT (adjusted OR = 8.44, *p* = 0.018), and RE (adjusted OR = 11.4, *p* = 0.009). For the SLEDAI and SDI scores, significant associations were found between their scores and lower PF (adjusted OR = 6.98, *p* = 0.001), lower RP (adjusted OR = 5.19, *p* = 0.009), and lower BP (adjusted OR = 3.26, *p* = 0.017) (Table 2). Additionally, by regression analysis, the MH domain of the SF-36 was significantly associated with the anxiety level in SLE patients (*p* = 0.001).

## 4. Discussion

SLE is a chronic, multisystem autoimmune disorder that provokes inflammation in various parts of the body [27]. The psychological and psychological activities of patients with SLE are known to affect their QoL. Psychological assessment and QoL assessment for patients with SLE are completed to improve or alleviate patients’ anxiety, thereby improving patients’ awareness of life quality. For this reason, it is frequently useful to understand each patient’s actual needs and expectations. In addition, some research results have shown that greater self-confidence and greater effectiveness in disease management will benefit patients’ mental health [28]. Notably, the assessment of QoL used in the present study is considered to be an effective and reliable tool for health outcomes in patients with SLE. Increasingly, even though QoL indicators are used to assess patients’ comprehensive experience of the disease and treatment, there is no dedicated SLE survival scale. In the present study, the proportion of female patients was much higher than that of male patients, which is consistent with the literature [27]. In addition, we found that education level and income were limited for most of the SLE patients, most of whom had graduated from high school or technical secondary school and belonged to middle-income families. Patient age, education level, and duration of illness can all affect the scores on the assessment. We also found that the levels of daily stress in our patients were comparable with those among the general population [29]. We found that the MCS, MH, and BP occupied the dominant positions among the SF-36 domains. In addition, the MH domain of the SF-36 was significantly associated with anxiety in SLE patients.

SLEDAI is an indicator widely used in clinical practice to reflect the disease activity of SLE. It reflects the degree of damage to patients’ organs and the severity of the disease. A higher score indicates more severe disease activity and worse health. The results of this study revealed differences in the level of anxiety among patients with different disease activity levels. In general, with a more severe condition, the patient experienced greater anxiety. One contributing factor is that the cost of treatment is high, and it will place a heavy burden on patients and their families, causing patients to be more likely to have negative emotions and increase anxiety. However, when the patients’ disease is inactive, the anxiety score was improved. Anxiety can also be caused by a patient’s lack of understanding of their condition and uncertainty about the future, which cause the patient to be concerned about their health and to seek good treatments. Of course, when the condition is inactive, patients will receive less care. If negative emotions are not addressed, the patient’s anxiety will increase. Our results also showed that the SDI score was correlated with the PF, RP, and BP on the SF-36, which is in contrast to the SLEDAI score for the physical domains of the SF-36. In addition, a previous study showed that disease activities in SLE patients showed close relationships with patients’ QoL [30,31,32], which is also demonstrated in the present study. Organ damage in SLE patients can obviously affect patients’ QoL [33]; however, contrary evidence has been obtained [33,34,35,36]. A previous study demonstrated that the inverse relationship between organ damage and QoL is mainly confined to the physical component of the SF-36 [31]. A possible explanation for the difference may be differences in several factors, such the regional differences, sex differences, differences in the disease activity metric used, etc. [33]. While our study supports the former results, a close relationship was found between organ damage and QoL. The reason may be that SLE patients become accustomed to having this disease and slowly understand the characteristics of the disease, making them able to properly accept the disease and the impact of the long-term treatment process on their life, which is also supported by a previous study [34]. In addition, chronic illness is certain to affect patients’ physical and mental health [35]. However, the SLEDAI and SDI scores were not associated with patients’ social activities, meaning they may have a lighter impact on the QoL of patients with SLE [37]. Therefore, all three aspects should be evaluated in SLE patients in order to obtain a complete clinical picture [34].

In the present study, we observed negative relationships between irregular sleep and the RP, VT, and RE scores in SLE patients. For most patients, sleep quality is a very important influencing factor, and one that has a greater influence on diseases with pain symptoms [38]. Physicians recognize sleep disturbance as a common complaint among SLE patients, and a previous study showed that more than 50% patients with SLE suffer sleep disturbance due to their disease, both in relation to mental stress and physical pain [39,40]. Moreover, poor sleep quality will worsen the patient’s negative emotions and clinical symptoms, which will not be conducive to overall treatment [41].

To date, a few studies on anxiety and QoL in SLE patients in China have been conducted. For example, Zhao et al. found a significant relationship between anxiety and body image disturbance (BID) in Chinese SLE patients [42]. Mok et al. conducted a large-scale questionnaire survey and analysis in SLE patients in southern China and found that the PF and MH domains occupied the foremost positions among all SF-36 domains [13]. Several possible reasons may explain the differences between our study and previous studies. The SLE patients in our study were from western regions of China, where anxiety is more prevalent than in the southern region [13]. In addition, the education level among SLE patients in western regions of China is generally lower than those in patients in the southern region. Most of the patients in the present study were from low- and middle-income families. Unfortunately, they may lack awareness of the disease and be more afraid of the treatment and its cost. Such fear may delay the time for medical treatment and prolong the illness, thus forming a vicious circle and affecting patients’ QoL. Therefore, we should pay greater attention to the treatment of patients in the western region of China.

The present study does have some limitations. The sample size was small, and the short investigation time limited analyses of factors such as disease manifestations and treatments. Despite these limitations, our study provides a preliminary understanding of the QoL of patients with SLE in western China. In future studies, we need to compensate for these shortcomings to obtain a more systematic and comprehensive understanding of SLE so that patients can be treated well.

## 5. Conclusions

The results of the present study highlight the need for more attention to be given to the psychological problems experienced by SLE patients during disease management and their connection with patients’ HRQoL. Additional research is needed to develop strategies caregivers can use to provide effective support as well as strategies for teaching SLE patients to recognize mental health problems and master self-management techniques that have long-term implications. Corresponding nursing measures must be applied to support both the physical and mental health of SLE patients, and it is also important that such measures be applied in the treatment of patients with mild SLE. A positive psychological state can promote patients’ active cooperation with treatments, thus supporting the rehabilitation process and improving the QoL of the patient.

## Figures and Tables

**Table 1:** Demographic data and disease characteristics of the SLE patients in this study.

Variables		n %	
Gender	Male	9	8.50
	Female	97	91.50
Age (years)	<18	10	9.43
	19–30	25	23.58
	31–40	18	16.82
	41–50	31	29.25
	51–60	14	13.21
	>60	8	7.55
Race	Han	103	97.17
	Others	3	2.83
Career	Worker	21	19.81
	Farmer	25	23.58
	Administrative staff	13	12.26
	Service industry	9	8.49
	Intellectual	7	6.60
	Others	31	29.25
Residence	Country	48	45.28
	City	58	54.72
Education	Primary school	11	10.38
	Junior middle school	22	20.75
	High school or technical secondary school	37	34.91
	Junior college	12	11.32
	Bachelors	18	16.98
	Masters or above	6	5.66
Marital status	Single	38	35.85
	Married	65	61.32
	Divorced	3	2.83
	Widowed	0	0
Income/person/ year	≤1000	17	16.04
	1000–3000	45	42.45
	3000–5000	31	29.25
	≥5000	13	12.26
Ability of financial situation to cover medical expenses	Enough	39	36.79
	Not enough	67	63.21
Medical expenses payment method	Rural cooperative medical service	48	45.28
	Health care in cities and towns	23	21.70
	Worker health	22	20.75
	Self-paying	13	12.26
	At state expense	0	0
Exercise	Never	30	28.30
	1–2 times/month	38	35.85
	1–2 times/week	21	19.81
	≥3 times/week	17	16.04
Faith	No	99	93.4
	Yes	7	6.6
Offspring	No	37	34.91
	Yes	69	65.09
Age at onset (years)	<18	10	9.43
	18–30	45	42.45
	31–40	26	24.53
	>40	25	23.58
Disease duration (years)	<1	26	24.52
	1–5	34	32.08
	6–10	21	19.81
	>10	25	23.58
Dosage of hormone (mg/d)	≤4	15	14.15
	5–8	35	33.02
	9–16	41	38.68
	>16	15	14.16
SLEDAI score	0–4	31	29.24
	5–9	34	32.08
	10–14	28	26.42
	≥15	13	12.26
Family history of illness	No	99	93.40
	Yes	7	6.60
Previous psychiatric symptoms	No	98	92.45
	Yes	8	7.55
Medication compliance	Worse	2	1.89
	Bad	4	3.78
	General	59	55.66
	Good	23	21.70
	Very good	18	16.98
Relapse	No	32	30.19
	Yes	74	69.81
Erythema	No	50	47.17
	Yes	56	52.83
Hair loss	No	25	23.58
	Yes	81	76.42
Dental ulcer	No	24	22.64
	Yes	82	77.36
Damage to other organs	No	39	36.79
	Yes	67	63.21
Arthralgia	No	55	51.89
	Yes	51	48.11
Knowledge of disease	Very familiar	35	33.02
	Some knowledge	63	59.43
	No knowledge	8	7.55
Worried about side effects	Not at all	3	2.83
	Not worried	6	5.66
	A little worried	43	40.57
	Worried	35	33.02
	Extremely worried	19	17.92
Sleep quality	Very good	6	5.66
	Good	11	10.38
	Neutral	53	50.00
	Bad	31	29.25
	Worse	5	4.72
Attitudes toward self-image	Not satisfied	41	38.68
	Neutral	54	50.94
	Satisfied	11	10.38
VAS score	0	23	21.7
	1–3	39	36.8
	4–6	28	26.41

**Table 2:** Logistic regression of contents of SF-36 on sleep quality, SLEDAI and SDI scores, and anxiety levels of SLE patients.

Independent Variables	Sleep Quality		SLEDAI and SDI Scores		Anxiety Level	
	*p*	OR (95% CI)	*p*	OR (95% CI)	*p*	OR (95% CI)
RP	0.011	8.32 (4.53–11.24)	0.011	7.59 (3.69–14.01)	0.011	2.59 (1.11–5.14)
VT	0.018	8.44 (3.57–10.22)	0.002	8.11 (5.82–18.35)	0.062	5.31 (1.33–7.22)
RE	0.009	11.4 (7.25–15.09)	0.030	7.76 (0.74–5.12)	0.057	4.55 (2.44–8.31)
PF	0.012	5.43 (2.42–7.77)	0.001	6.98 (40.25–10.28)	0.011	3.27 (1.25–8.58)
RP	0.034	6.32 (4.74–85.37)	0.009	5.19 (1.25–8.58)	0.019	1.95 (0.81–4.70)
BP	0.043	1.76 (0.74–3.56)	0.017	3.26 (1.55–5.53)	0.007	5.26 (3.03–11.11)
MH	0.001	4.67 (2.25–6.58)	0.001	7.21 (5.53–9.24)	0.001	8.21 (4.53–13.41)
